# PGASO: A synthetic biology tool for engineering a cellulolytic yeast

**DOI:** 10.1186/1754-6834-5-53

**Published:** 2012-07-27

**Authors:** Jui-Jen Chang, Cheng-Yu Ho, Feng-Ju Ho, Tsung-Yu Tsai, Huei-Mien Ke, Christine H-T Wang, Hsin-Liang Chen, Ming-Che Shih, Chieh-Chen Huang, Wen-Hsiung Li

**Affiliations:** 1 Biodiversity Research Center, Academia Sinica, Taipei, 115, Taiwan; 2 Genomics Research Center, Academia Sinica, Taipei, 115, Taiwan; 3 Department of Life Sciences, National Chung Hsing University, Taichung, 402, Taiwan; 4 Biotechnology Center, National Chung Hsing University, Taichung, 115, Taiwan; 5 Microbial Genomics, National Chung Hsing University, Taichung, 402, Taiwan; 6 Agricultural Biotechnology Research Center, Academia Sinica, Taipei, 115, Taiwan; 7 Department of Ecology and Evolution, University of Chicago, Chicago, IL, 60637, USA

**Keywords:** Consolidated bioprocess, Synthetic biology, Yeast, Cellulolytic enzymes, Bio-ethanol

## Abstract

**Background:**

To achieve an economical cellulosic ethanol production, a host that can do both cellulosic saccharification and ethanol fermentation is desirable. However, to engineer a non-cellulolytic yeast to be such a host requires synthetic biology techniques to transform multiple enzyme genes into its genome.

**Results:**

A technique, named Promoter-based Gene Assembly and Simultaneous Overexpression (PGASO), that employs overlapping oligonucleotides for recombinatorial assembly of gene cassettes with individual promoters, was developed. PGASO was applied to engineer *Kluyveromycesmarxianus* KY3, which is a thermo- and toxin-tolerant yeast. We obtained a recombinant strain, called KR5, that is capable of simultaneously expressing exoglucanase and endoglucanase (both of *Trichodermareesei*), a beta-glucosidase (from a cow rumen fungus), a neomycin phosphotransferase, and a green fluorescent protein. High transformation efficiency and accuracy were achieved as ~63% of the transformants was confirmed to be correct. KR5 can utilize beta-glycan, cellobiose or CMC as the sole carbon source for growth and can directly convert cellobiose and beta-glycan to ethanol.

**Conclusions:**

This study provides the first example of multi-gene assembly in a single step in a yeast species other than *Saccharomyces cerevisiae*. We successfully engineered a yeast host with a five-gene cassette assembly and the new host is capable of co-expressing three types of cellulase genes. Our study shows that PGASO is an efficient tool for simultaneous expression of multiple enzymes in the kefir yeast KY3 and that KY3 can serve as a host for developing synthetic biology tools.

## Background

Among the current approaches to cellulosic ethanol production, consolidated bioprocessing (CBP) is most preferred because of its simplicity and potential low cost [1]. To achieve CBP, a microbe that can carry out cellulase production, hydrolysis, and fermentation in a single process is needed. Currently, however, there is no single microbe available for doing CBP efficiently. Although *Saccharomyces cerevisiae* has been considered the best ethanol producer from hexose sugars, its genome lacks genes for cellulolytic enzymes. Thus, there have been efforts to introduce cellulase genes into *S. cerevisiae*[2,3]. Previously, we made attempts to improve the signal peptide for secretion or to reduce the glycosylation strength of *S. cerevisiae*, so that we could over-express cellulase genes of other fungi in *S. cerevisiae*. Unfortunately, most of the expressed proteins were either non-functional or could not be efficiently secreted out of the cell (data not shown). Recently, we isolated a kefir yeast, *Kluyveromyces marxianus* KY3 (data not shown), that has the potential to serve as a host for bioethanol production and a biorefinery platform, because the strain has broad substrate spectrum, including both hexose and pentose sugars, and produce valuable flavor byproducts such as 2-phenylethanol. The strain also shows resistant to inhibitors generated from chemical pretreatment of lignocellulose and is heat-tolerant [4,5]*.* Moreover, many genetic and genomic tools such as those developed for *K. lactis*[6,7] are applicable to KY3.

Recently, synthetic biology has been recognized as a powerful approach for the design and construction of new biological systems. Although cloning tools such as the Univector plasmid-fusion system [8], ligase-free [9] or ligation-independent cloning (LIC) [10], and the Gateway cloning system [11,12] have been developed and widely adopted, a technique that can assemble multiple genes in a desired order in a single step and integrate the large DNA piece into a genome is highly desirable. To achieve this goal, various techniques have been developed to enable the assembly of several genes or DNA modules into a larger construct, including chain reaction cloning [13], the OGAB method [14], DNA assembler *in vivo*[15], USER cloning [16], MAGIC [17], SLIC [18], In-Fusion Clontech [19], Illegitimate recombination [20], Circular polymerase extension cloning [21], and one-step assembly in yeast [22,23]. These methodologies are based mainly on historically well-characterized hosts, such as *Escherichia coli, Bacillus subtilis* and *S. cerevisiae,* whereas newly discovered organisms with great characteristics for bioprocessing are still in need of synthetic biology tools.

The synthetic biology technique we developed in this study relies on homologous recombination, which is responsible for a number of important transformation processes of microorganisms and is very useful for the generation of host cells for both cloning and expression of heterologous genes. Several transformation systems have been developed for use with *S. cerevisiae* by episomal plasmids and integrating plasmids with foreign DNA fragments [15,24,25]. In a previous study, an extending homologous recombination approach was demonstrated by assembling a megaplasmid from multiple overlapping fragments in a single step in *S. cerevisiae*[22]. Moreover, episomal plasmid and integrating plasmid transformation studies on other yeast species, such as *K. lactis*, have also been reported [6,7,26]. Although non-homologous end-joining (NHEJ) is a common phenomenon in fungi and serves as a transformation method in *K. marxianus*[20,27], the homologous recombination strategy has several advantages for genetic engineering in fungi, such as ordered multiple gene assembly in one step [22,23]. Compared to NHEJ, this strategy is simpler for controlling the copy numbers of interested genes in a genome, and for targeting specific genes for insertion or disruption.

The technique we developed is called “Promoter-based Gene Assembly and Simultaneous Overexpression (PGASO)”. PGASO has four advantages for genome engineering: (1) Multiple genes can be transformed into a genome in a single step; (2) specific upstream promoter sequences can be used in the gene assembly in a predesignated order without linker sequence; (3) each gene cassette has a unique promoter, so that its expression level can be adjusted; and (4) PGASO can be applied to a host that can undergo homologous recombination. As an example of application, we applied PGASO to integrate five gene cassettes in a predesignated order into a specific site in the genome of *K. marxianus* KY3 in a single step. The five genes include one reporter gene, one selectable marker gene, and three different essential cellulase genes for cellulose saccharification. The purpose of this construct is to engineer *K. marxianus* KY3 into a host for cellulose saccharification and ethanol fermentation in one step. This is the first example of multi-gene assembly in a yeast species other than *S. cerevisiae*.

## Results and discussion

### The technical concept

**Figure 1:**
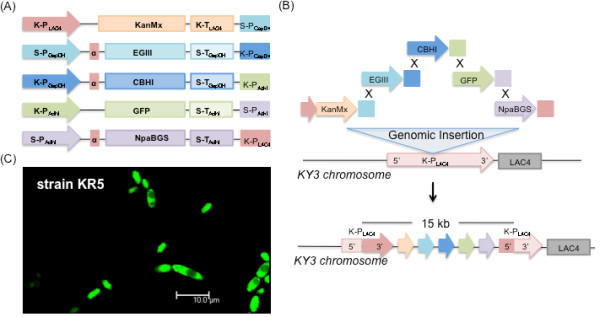
**Genomic integration of five gene cassettes into KR3.** (**A**) Each of the five gene cassettes contains an independent promoter, alpha factor from *K. lactis*, a gene coding region, a terminator, and a 46 bp fragment homologous to its neighboring cassette. The KanMX, EGIII, CBHI, GFP, and NpaBGS gene cassettes are shown in orange, blue, dark blue, green, and purple, respectively. (**B**) The gene cassettes assembled in the predesignated order, Kan-EGIII-CBHI-GFP-NpaBGS. (**C**) Fluorescence microscopy photograph of the genetically engineered strain, KR5.

We developed the Promoter-based Gene Assembly and Simultaneous Overexpression (PGASO) technique to insert multiple gene cassettes in a predesignated order into the genome of a cell. Each gene cassette contains 2 parts: (1) the gene sequence linked, at the 5’ end, to a promoter sequence, and (2) a sequence at the 3’ end of the gene cassette that is identical to the 5’ end of the adjacent cassette (Figure 1). A portion of the 5’ end of the promoter sequence for the first gene cassette and a portion of the 3’ end of the last gene cassette are homologous to a predetermined site in the host genome in order to facilitate site-specific insertion. The promoter sequence in a gene cassette should be different from that of all other gene cassettes. The sequence at the 3’ end of a gene cassette, however, should be homologous to a portion of the promoter sequence in the adjacent downstream gene cassette. When the gene cassettes are introduced into the cells, they join together in the predesignated order via homologous recombination between the pairs of overlapping and promoter sequences, and they are inserted into the genome via homologous recombination at the promoter sequence of the first and the 3’ end of the last gene cassette. To examine the insertion probability of a non-specific gene fragment in our experiment, the single KanMX gene cassette (kan) that possessed a single homologous recombination site in the *K. marxianus* KY3 genome was transformed into the host. The same molar ratio of a positive control vector with the KanMX gene that possessed two recombination sites in the KY3 genome was also transformed as the control strain (NC). The transformation efficiencies of kan and NC were calculated by counting the colonies on the plates with G418, and the ratio was 1:93. This benchmark test showed the potential of transformation via homologous recombination in *K. marxianus* KY3. In this study, our use of the specific 5’-upstream region of the promoter as the specific homologous recombination sites, requiring no linkers, is the first such application in a yeast other than *S. cerevisiae*. PGASO is potentially applicable to any host that can undergo homologous recombination.

### Insertion of five gene cassettes

To confer the hydrolysis ability of *K. marxianus* KY3 on higher order carbon sources such as cellulose, three cellulase genes, a selection marker gene and a reporter gene were introduced into the KY3 genome. The three cellulase genes were a beta–glucosidase gene (NpaBGS), originally found in a cow rumen fungus [28], and two *T. reesei*cellulase genes (i.e., an endo-glucanase gene (EGIII) and an exo-glucanase gene (CBHI)). The neomycin phosphotransferase gene essential for G418 resistance (Kan MX) was used as a marker gene for clone screening. The green fluorescent protein (GFP) gene was employed as a promoter reporting system and as a biosensor to monitor cell state; this gene can be readily replaced by a cellulase gene to increase the glucanase activity. The five genes were assembled as one single cassette for genetic manipulation via recombinatorial insertion. To reduce unexpected recombination events in multi-gene integration into similar regions, various unique heterologous promoters were used. To express these cellulases in the same culture for multiple enzyme reactions, several constitutive promoters were chosen for the gene cassettes. These promoters included GapDH (ScPGapDH) and ADHI (ScPADHI) from *S. cerevisiae* and Lac4 (PLac4), GapDH (KlPGapDH) and ADHI (KlPADHI) from *K. lactis*, which showed only 40-55% sequence identity among each other in the 5’ upstream regions.

We achieved the transformation of five gene cassettes in a single step into the genome of *K. marxianus* KY3 using the PGASO method. The order and composition of the resulting single five-gene cassette was as follows. The first gene was a selection marker gene (the KanMX gene, 810 bp) linked with a portion of PLac4 promoter. The second gene, an endoglucanase gene (the EGIII gene, 1449 bp), was linked with the KlPGapDH promoter. The third gene, an exoglucanase gene (the CBHI gene, 1749 bp), was driven by the KlPGapDH promoter. The fourth gene contained a reporter gene (the GFP gene, 720 bp) and was driven by the ScPADHI promoter. The last gene, a beta-glucosidase gene (the NpaBGS gene, 2526 bp), was linked with the KlPADHI promoter. These five gene cassettes were prepared by PCR, with a 46 bp overhanging sequence to the 5’ end of each promoter and a 46 bp overhanging sequence to the 3’ end of the terminator region (Figure 1A). The overhangs were designed to facilitate homologous recombination, because the 5’ end of each fragment overlaps with the 3’ end of its 5’ upstream neighbor; the 5’ overhang of the first cassette (the KanMX gene) and the 3’ terminal on the last cassette (the NpaBGS gene) overlap with the Lac4 promoter region in *K. marxianus* KY3 (Figure 1B). Consequently, the five gene cassettes, each with an independent promoter, alpha factor from *K. lactis*, gene coding region, and terminator, were assembled in the designed order as “Kan-EGIII-CBHI-GFP-NpaBGS” with a total DNA fragment length of 14,877 bp, which was then integrated into the Lac4 promoter region of *K. marxianus* KY3 via a single-step genome recombination (Figure 1B). A transformed strain, the *K. marxianus* KR5 strain, was selected with G418 resistance; the activation of green fluorescent protein via promoter ScPADHI was confirmed by fluorescence microscopy (Figure 1C).

**Figure 2:**
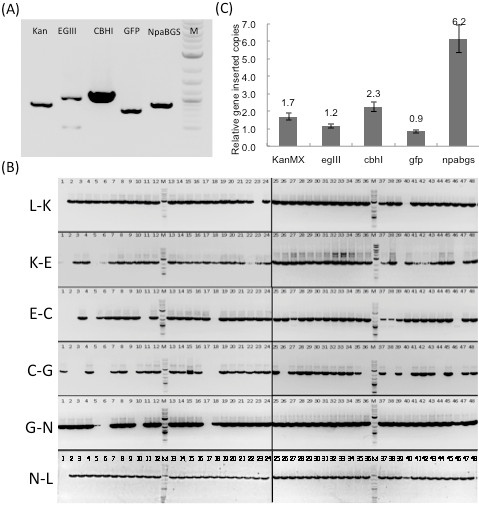
**Gene insertion confirmation and copy-number quantification.** (**A**) The five-gene insertion was confirmed by PCR with five pairs of gene specific internal primers. The PCR products revealed five specific bands: 810 bp (kan), 1012 bp (egIII), 1068 bp (cbhI), 750 bp (gfp), and 896 bp (NpaBGS). (**B**) The order of the gene cassettes was confirmed by PCR with five internal primer pairs. The PCR products resulted in six specific amplicons. (**C**) The relative ratios of the inserted gene copy numbers relative to the alg9 gene in KR5.

The five-gene insertion in KR5 was confirmed by PCR using five pairs of gene-specific internal primers (Table 1); the PCR products could be resolved in five specific bands (Figure 2A). The multi-gene transformation efficiency of the KY3 host is high, as all the selected 48 colonies had the five heterologous gene cassettes. The transformation efficiencies of the benchmarks, kan and NC, and KR5, were calculated by counting the colonies on the G418 plates, and their ratio was 1:93:24. To verify that these gene cassettes were assembled in the correct order, five internal primer pairs spanning the gap regions of each cassette were designed (Table 1). The PCR products showed in five specific bands, L-K, K-E, E-C, C-G, G-N, and N-L with correct sizes, 2400 bp, 2293 bp, 1712 bp, 2273 bp, 1905 bp, and 2575 bp, respectively (Figure 2B). The one-step multi-gene fragment assembly method has thus been successfully demonstrated in KR5. The accuracy of assembly was also high, as an average of 62.5% colonies were found to have a five gene cassettes assembly with the predesigned order. To determine the relative copy number of these five genes in KR5, the genomic DNA was isolated from KR5 and from the control strain NC for quantitative PCR analysis, using the 5 gene-specific primer sets (Table 1). The ratios of the inserted copy-numbers of the five genes in the constructed KR5 isolates relative to the indigenous alg9 gene were approximately 1.7 (kan), 1.2 (egIII), 2.3 (cbhI), 0.9 (gfp) and 6.2 (npabgs) (Figure 2C). Although the probability of non-specific insertion was not high, these data suggested that the unequal gene copy numbers might be caused by non-specific gene insertions or incomplete gene cassette assembly. Similar results were obtained using the indigenous actin gene as the reference gene (data not shown).

**Table 1:** The primer pairs used in the PGASO construction

**Primer name**		**Sequence**
**Cassette construction**		
Lac4-KanMx	Kl-PLac4-3’End-F	5’-TAGGGCCTGTTTGGCCTCCCGCGGGGATC-3’
	Kl-LAC4_46bpScPGap_Dra3_R	5’-TAGCACTCAGTGATTATTTACGTATTCTTTGAAATGGCAGTATTGATAATGATAAACTTATACAACATCGAAGAAGAGTC-3’
ScGapDH-EgIII	ScPGapDH-F-BglI	5’-TAGGCCATGACGGCAGTTTATCATTATCAATACTGCC-3’
	AFEgIII_ScPGapDH_R	5’-GTAGAGAATTTCATTTTTTTGTTTGTTTATGTGTGTTTAT -3’
	ScPGapDH_AFEgIII_F	5’-ATAAACACACATAAACAAACAAAAAAATGAAATTCTCTAC-3’
	ScTTGap_EgIII_R	5’-AAGATTTAAAGTAAATTCACGCGGCCGCCTACTTTCTTGCGAGACACG -3’
	EgIII_ScTTGap_F	5’-CGTGTCTCGCAAGAAAGTAGGCGGCCGCGTGAATTTACTTTAAATCTT-3’
	ScTTGap_Kl_PGapDH_R	5’-CTTTTCCATTTGCCTTCGCGCTTGCCTGTACGGTCGTTACCATACTTGGCGGAAAAAATTCATTTG -3’
KlGapDH-CBHI	Kl-PGapDH-F	5’-AGTATGGTAACGACCGTACAGGCAA-3’
	AFCBHI_KlPGapDH_R	5’-GTAGAGAATTTCATTTTTTTTGTGTAATATTCTTTTTTTT-3’
	KlPGapDH_AFCBHI_F	5’-AAAAAAAAGAATATTACACAAAAAAAATGAAATTCTCTAC -3’
	ScTTGap_CBHI_R	5’-AAGATTTAAAGTAAATTCACGCGGCCGCTTACAGGCACTGAGAGTAGT -3’
	CBHI_ScTTGap_F	5’-ACTACTCTCAGTGCCTGTAAGCGGCCGCGTGAATTTACTTTAAATCTT -3’
	ScTTGap_Kl_PADHI_R	5’-TGGTAACGACCGTACAGGCAAGCGCGAAGGCAAATGGAAAAGCTGGTGGCGGAAAAAATTCATTTG-3’
KlADHI-GFP	Kl-PADHI-F	5’-CCAGCTTTTCCATTTGCCTTCGCGCTTGCC-3’
	GFPKLADHI-R	5’-TCCTCGCCCTTGCTCACCATTTTATCTTTTTTTAGTATAGAGT-3’
	KLADHIGFP-F	5’-ACTCTATACTAAAAAAAGATAAAATGGTGAGCAAGGGCGAGGA-3’
	ScTTGap_46bpScPADHI_CGA-BglI_R	5’-TAGGCCGTCGTGGCATGTATGGGTTTGGTTGCCAGAAAAGAGGAAGTCCATATTGTACAC-3’
ScADHI-NpaBGS	ScPADHI_CGA-BglI_F	5’-TAGGCCACGACGGCGTGTACAATATGGACTTCCTCTTTTC -3’
	NpaBGS-BglII-F	5’-ACGAGATCTAAAAAAATGAAATTCTCT-3’
	NpaBGS-SmaI-R	5’-TATCCCGGGTTAGTAAAGTTTGTAAGC-3’
	Kl-PLac4 -5’End-R-SfiI	5’-AGGGCCAAGAAGGCCAGCCGCGGAAATTTAGGAATTTTAAAC-3’
**Checking primer**		
Kan	Kan-BglII-F	5’-AAAAAGATCTGCCACCATGGGTAAGGAAAAGACTC-3’
	Kan-XbaI-R	5’-AAAAATCTAGATTAGAAAAACTCATCGAGCAT-3’
EgIII	EgIII-1084 F	5’-GACATGTGCCAGCAAATCCAATATC-3’
	ScTTGap_Kl_PGapDH_R	5’CTTTTCCATTTGCCTTCGCGCTTGCCTGTACGGTCGTTACCATACTTGGCGGAAAAAATTCATTTG-3’
CBHI	Kl-PGapDH-F	5’-AGTATGGTAACGACCGTACAGGCAA-3’
	CBHI-218R	5’-AAGTGTTGCCATCGTAGCAGTTCGT-3’
GFP	GFP-BglII-F	5’-ACGAGATCTATGGTGAGCAAGGGCGA-3’
	GFP-SmaI-R	5’-TATCCCGGGTTACTTGTACAGCTCGTCCA-3’
NpaBGS	NpaBGS-1422-F	5’-TCCAGGTCCAGTTAATGTTCCATTC-3’
	NpaBGS-SmaI-R	5’-TATCCCGGGTTAGTAAAGTTTGTAAGC-3’
**Internal primer**		
amplicon L-K	Lac4-Primer1	5’-ACACACGTAAACGCGCTCGGT-3’
	Kan-126R	5’-TACAATCGATAGATTGTCGCACCTG-3’
	Kan-673 F	5’-CAGGATCTTGCCATCCTATGGAACT-3’
amplicon K-E	EgIII-528R	5’-TACTTGGAAATGCTCGTGGAATCAA-3’
amplicon E-C	EgIII-1084 F	5’-GACATGTGCCAGCAAATCCAATATC-3’
	CBHI-218R	5’-AAGTGTTGCCATCGTAGCAGTTCGT-3’
amplicon C-G	CBHI-585 F	5’-CGATCTGAAGTTCATCAATGGCCAG-3’
	GFP-150R	5’-GTGCAGATGAACTTCAGGGTCAGCT-3’
amplicon G-N	GFP-492 F	5’-GAACTTCAAGATCCGCCACAACATC-3’
	NpaBGS-403R	5’-CACATTCACCAACATAGAATGGATC-3’
amplicon N-L	NpaBGS −1422 F	5’-TCCAGGTCCAGTTAATGTTCCATTC-3’
	Lac4-3'-436-R	5’-ACTCTACATGCGACTTGGAAGGC-3’
**UPL system QPCR primer**		
Kan	Kan-UPL#144 F	5’- AGACTAAACTGGCTGACGGAAT-3’
	Kan-UPL#144R	5’- CATCAGGAGTACGGATAAAATGC -3’
EgIII	EgIII-UPL#77 F	5’- TGGCTCCGACAGAACAATC -3’
	EgIII-UPL#77R	5’- GTCTTGTATGCAGGACTGAACG -3’
CBHI	CBHI-UPL#77 F	5’- ACATCAAGTTCGGACCCATT-3’
	CBHI-UPL#77R	5’- GGTAGGTCCGGGAGAGCTT-3’
GFP	GFP-UPL#148 F	5’- TCTATATCATGGCCGACAAGC-3’
	GFP-UPL#148 F	5’- GTTGTGGCGGATCTTGAAGT-3’
NpaBGS	NpaBGS-UPL#150 F	5’- GAAGCTGTAATGGAAGAAGATGG-3’
	NpaBGS-UPL#150R	5’- CTGGGAATGAAAGGAAAATCAT-3’
Alg9	ALG9-UPL#151 F	5’- GTGGGTCTATACCACGTCTCATC-3’
Actin	ALG9-UPL#151R	5’- TCCAAATATAACGAATTTAAGCAACTT-3’
	ACTIN-UPL #9 F	5’- GCGTAGATTGGAACAACGTG-3’
	ACTIN-UPL #9R	5’- AGAACTACCGGTATTGTGTTGGA-3’

Our study shows that assembling specific sequences using overlapping fragments [22] is feasible and that PGASO can be tailored for various purposes via promoter design. Although we obtained a transformant (KR5) with the specified order of gene cassettes, the strain also contained some unexpected insertions resulting from the NHEJ pathway [20], especially the last gene cassettes. To increase the gene targeting insertion in the yeast genome, longer recombination fragments were employed at the border of the first and the last gene cassette. In transformants, a higher number of copies was found in both the first and the last gene cassette. These unanticipated insertions could be avoided by reducing the NHEJ effect. To reduce NHEJ, we note that inhibition of the DNA double-strand break (DSB) repair system was found to hinder the random gene integration process, resulting in a higher gene targeting efficiency by homologous DNA [27]. This may the next step to improve our PGASO system.

### Expression of the five heterologous promoters

**Figure 3:**
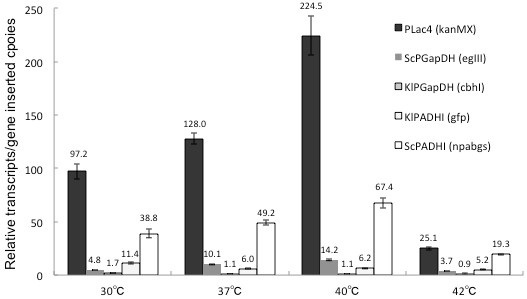
**The quantitative PCR analysis of the five gene cassettes in KR5.** The relative ratios of the five promoter transcripts are shown in comparison to the alg9 gene in KR5 at different temperatures. These ratios are normalized by the respective gene cassette insertion copy number estimates.

To analyze the gene expression profiles of the five heterologous promoters in KR5, KR5 cells were incubated at 30°C, 37°C, 40°C, and 42°C. Total RNA under each of these conditions was isolated from KR5 for quantitative PCR analysis, and alg9 was employed as the reference gene. After normalizing by the inserted gene copy-number, the expression levels of the five promoters, expressed as a multiple of alg9 expression, were 97.2 (PLac4), 4.8 (ScPGapDH), 1.7 (KlPGapDH), 11.4 (KlPADHI) and 38.8 (ScPADHI) at 30°C (Fig. 3). These data indicated that the PLac4 and ScPADHI promoters were stronger than the ScPGapDH, KlPGapDH, and KlPADHI promoters in KR5 in all of the experimental conditions used. When the incubation temperature of KR5 was raised to 37°C and 40°C, the expression level of PLac4, ScPGapDH and ScPADHI were simultaneously raised. On the other hand, incubation at 42°C was not a favorable condition for the expression of all five heterologous promoters in KR5. The transcription profiles revealed that 40°C might be an optimal gene expression condition for the KR5 system using these five promoters.

These observations also indicate unequal gene copy numbers and transcript abundances among the five genes. These inequalities might have been caused by both non-specific gene insertions and differences in transcriptional efficiency among the promoters. A comparison of transcript abundances suggests that in KR5 the GapDH and AdhI promoters from *K. lactis* are inherently weaker and the AdhI promoter from *S. cerevisiae* is stronger than the other constitutive promoters used in this study. All regulatory elements (promoters) used here were derived from strong constitutive genes associated with yeast-specific metabolic pathways. The use of this type of promoter is advantageous, as the engineered strains can be driven under the normal growth, on different carbon sources, or under a high cell density immobilization condition. Moreover, the wide spectrum of induction strengths observed in different promoters may be drawn upon to devise efficient gene expression systems for optimal enzyme-cocktails or to study gene regulation in yeast.

### Characterization of the secreted cellulases of KR5

**Figure 4:**
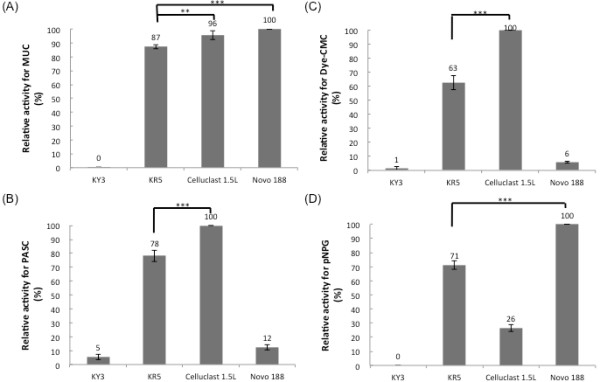
**Cellulolytic enzyme assays of*K. marxianus*transformants. The relative activities were conducted using (A) MUC, (B) Dye-CMC, (C) PASC, and (D) pNPG as the substrate, respectively.** The commercial cellulolytic enzyme mixture kits with 0.5 unitCelluclast 1.5 L and 1 unit Novozyme 188 were used as benchmarks. The protein concentration of the supernatant of the *K. marxianus* transformants cultures were 1.3 mg/ml. *: P < 0.05 (significant), **: P < 0.01; ***: P < 0.001; N.S., non-significant.

To quantify the secreted cellulase activities, the supernatant of KR5 was harvested for analysis without protein purification. The commercial cellulolytic enzyme mixture kits Celluclast 1.5 L and Novozyme 188 were used as the benchmarks; the supernatant with KR5 secreted cellulases and diluted commercial enzymes were estimated using an equal MUC activity which represented the total glucanase activity. The MUC activity assay was performed with MUC as the substrate, and the results indicated that the MUC activity in the supernatant of KR5 was equivalent to those of 0.5 unit of Celluclast 1.5 L and 1 unit of Novozyme 188, and higher than that of the control strain (Figure 4A). The glucose assay indicated significantly improved digestion of PASC by KR5; the activity was up to 80% of that of the 0.5 unit of Celluclast 1.5 L (Figure 4B). The activity assay with Dye-CMC as the substrate suggested that the endo-glucanase activity in the supernatant was significantly improved due to the EGIII secreted by KR5; the activity was 60% of the 0.5 unit of Celluclast 1.5 L (Figure 4C). The activity assay with pNPG as the substrate showed that the beta-glucosidase activity of NpaBGS in the supernatant of KR5 was higher than that of the control strain, and the activity was nearly 80% of the 1 unit of Novozyme 188 (Figure 4D). These data demonstrated successful co-expression of the exogenous fungal genes and secretion of their gene products without any significant post-translational modification problems.

Successful protein production in a heterologous host at a commercial scale often requires the regulation of the timing and the expression level of the cloned gene(s). The long culturing-time required by fungi is a current bottleneck of traditional enzyme purification technologies, such as Celluclast 1.5 L from *Trichoderma* and Novo188 from *Aspergillus*. The faster growth rate of *K. marxianus* makes it more desirable for commercial enzyme production. In addition to efficient expression vectors and transformation protocols, heterologous gene expression also depends on a signal sequence to direct the secretion of the synthesized protein to the extracellular environment. In this study, we have used the promoters described above and the signal sequence of the *K. lactis* alpha-mating factor to express and secret the heterologous cellulases in KR5, at a much higher efficiency than the classical *S. cerevisiae* system. Furthermore, the new host strain *K. marxianus* KR5 is not only naturally competent to secrete enzymes, but also efficient for combining different enzyme systems for downstream processing of low-cost industrial enzymes.

### Sugar utilization and ethanol production assay

**Figure 5:**
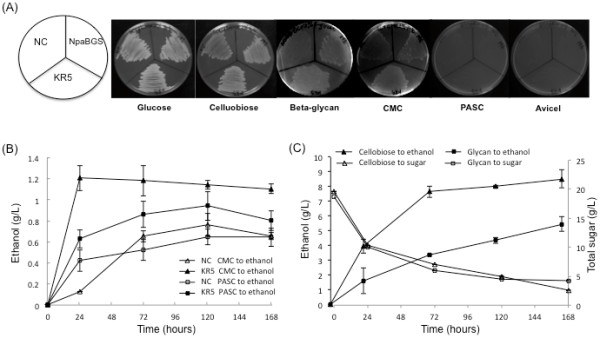
**The growth patterns with different carbon sources and simultaneous saccharification and fermentation ability assays.** Yeast strains in **(A)** YP medium plates with different carbon sources, **(B)** YP liquid medium containing 2% CMC or 2% PASC or **(C)** 2% cellobiose or 2% beta-glycan as the sole carbon source by strains NC (vector for control), NpaBGS (KY3-NpaBGS), and KR5. The total sugar concentration was measured as glucose equivalent by the phenol-sulfuric acid method.

Several types of cellulose were tested in this study to determine the carbon source utilization and ethanol fermentation abilities of KR5, KY3-NpaBGS, which is a KY3 strain transformed with the NpaBGS gene, and the control strain NC. All three strains are capable of utilizing glucose and cellobiose for growth, and KY3-NpaBGS can grow on beta-glycan substrate, but only KR5 can significant assimilate beta-glycan and CMC (Figure 5A). To examine the SSF ability of KR5, fermentation was performed in the YP medium containing cellobiose, beta-glycan, CMC or PASC as the sole carbon source. After cultivation of cells in YPD medium for 24 h at 30°C, the cells, which had an O.D. of 20, were harvested for subsequent inoculum. As shown in Fig. 5, KR5 could use cellobiose, beta-glycan, CMC or PASC as the sole carbon source for fermentation. When cellobiose was used as the carbon source, KR5 produced 8.5 g/L ethanol with a 93% conversion ratio in 168 h at 37°C (Figure 5A). This efficiency of cellobiose utilization is as good as its glucose utilization, When 2% beta-glycan was the sole carbon source, KR5 produced 5.4 g/L ethanol with a 74% conversion ratio in 168 h at 37°C (Figure 5B). These data indicated that KR5 could express cellulolytic enzymes and directly produce ethanol from cellulosic materials. The CMC and PASC assimilation abilities were only moderately increased compared to the control strain (Figure 5C). However, it is unclear why KR5 could not efficiently produce ethanol from CMC and PASC. Prior research reported that *K. marxianus* might not utilize carboxymethyl glucose, which is released from carboxymethyl cellulose and may inhibit cell growth [29]. It has been reported that while *K. marxianus* could be transformed with different cellulase genes, a higher endoglucanase enzyme expression level than other enzymes could be critical for cellulolytic reactions [30,31]. Therefore, in future work we will construct new KY3 strains that can efficiently convert cellulose into component sugars by integrating more cellulolytic genes and by optimizing multiple gene expression ratios via the PGASO method. 

*K. marxianus* has a number of advantages, such as heat and toxin tolerance, over the model organisms *K. lactis* and *S. cerevisiae*[5]*.* PGASO can be applied to facilitate enzyme combinations or to construct desired pathways in *K. marxianus* KY3. The wide spectrum of induction strengths observed in different promoters may be drawn upon to devise efficient gene expression systems for optimal enzyme-cocktails or to study gene regulation in yeast. Furthermore, PGASO is potentially applicable to any host that can undergo homologous recombination. The potential to control the expression ratios of different enzymes is a sought-after feature for many applications. Our study has demonstrated a success in the development of this yeast strain as a new host for heterologous protein production and as a cell factory for enzyme production.

## Conclusion

Our use of the specific 5’-upstream region of the promoter as the specific homologous recombination sites, requiring no linkers, is the first such application in a yeast other than *S. cerevisiae*. In this study, we have developed a new synthetic biology tool, PGASO, and used it to engineer a new yeast host, KR5, to express three types of highly efficient cellulases for biomass conversion. KR5 showed a significant improvement in lignocellulosic polysaccharide utilization and ethanol conversion. Our study demonstrated a successful development of this yeast strain as a new host for heterologous protein production and as a cell factory for enzyme production. It also showed that *K. marxianus* KY3 is a good host for multi-gene assembly and genome engineering via the synthetic biology approach. The potential of PGASO to control the expression ratios of different enzymes is a sought-after feature for many applications.

## Methods

### Multiple-gene cassette construction

An application of PGASO to assemble five gene cassettes in a predesignated order is illustrated in Figure 1. In the first gene cassette, the KanMX gene and the *Lac*4 promoter fragment from pKlac2 vector (*K. lactis* Protein Expression Kit, New England Biolabs) were amplified and assembled into a fragment with the Lac4-KanMX primer pairs. The coding regions of the second and the third gene cassettes, the EGIII gene and the CBHI gene, were amplified from the cDNA of *T. reesei* and assembled with the ScGapDH promoter and KlPADHI promoter region by the ScGapDH-EgIII and the KlGapDH-CBHI primer pairs via fusion PCR, respectively. The fourth gene cassette with the gfp gene was constructed using the KlADHI-GFP primer pairs. The fifth gene cassette with the NpaBGS gene [28] and a ScADHI promoter were respectively amplified and constructed using the ScADHI-NpaBGS primer pairs. Consecutive gene cassettes containing overlapping 46 bp regions on the border are used for recombinatorial gene assembly. The PCR to check the assembly was conducted using the TaKaRa Ex Taq system and the primer pairs used are listed in Table 1. The reaction mixture contained 0.2 mM of each primer, 0.25 mM of each deoxynucleoside triphosphate, 1x PCR buffer with 2 mM MgCl_2_, 2 μL of DNA and 2.5 U of Ex Taq DNA polymerase. PCR reaction was carried out at 94°C for 1 min, touchdown annealing temperature from 58°C to 53°C for 1 min, and 72°C for optimized period for 10 cycles.

### Yeast transformation and clone screening

The cells were incubated in 5 ml YPD medium (1% BactoDifco-Yeast Extract, 2% BactoDifco-Peptone, 2% Merck-D(+)-Glucose) at 30°C, shaking at 200 rpm for 16 hr. To express heterologous enzymes in KY3, we followed the transformation method for *K. lactis* (Sánchez et al., 1993). The target DNA fragments in a 5 μg volume with an equal molar ratio of each fragment were mixed with 40 μl of competent cells. The electroporation was performed (1.0 kV, 400 Ω, and 25 μF capacitance) using a BioRad system (GenePluserXcell TM, Bio-Rad, Hercules, CA) with an aluminum cuvette (2 mm). The cells were spread onto YPG plates (1% BactoDifco-Yeast Extract, 2% BactoDifco-Peptone, and 2% Merck-galactose) containing G418 (200 μg/mL). To confirm the presence of each fragment, each isolated colony was digested in QucikExtract^TM^ DNA Extraction Solution (EPICENTRE, Madison, Wisconsin) to remove yeast cell wall and was then examined by PCR with gene specific checking primers (Table 1). Moreover, to verify that these gene cassettes were inserted into the correct position of the KR5 genome and assembled in the correct order, specific internal primer pairs of gaps of each cassette were designed and confirmed by PCR (Table 1). These clones were further examined under a bright field microscope with phase contrast and fluorescence with a GFP filter, and photographed by a confocal microscope and single molecule detection system (Leica TCS-SP5-MP-SMD, Germany).

### Quantitative PCR analysis

The cells of each isolate were incubated at 30°C, 37°C, 40°C, and 42°C with 200 rpm for 16 hr. The genomic DNA was purified from yeast cells using a DNA Isolation Kit III (DNA Isolation Kit III, Roche). The template mRNA was each purified from yeast cells using RNeasy Protect mini kits (High Pure RNA Isolation Kit, Roche). The cDNA synthesis was conducted using a reverse transcription kit (SuperScript^TM^ II kit, Invitrogen). The relative quantification of each gene was carried out via the Universal Probe Library Set (LightCycler® 480 Probes Master, Roche) with a specific primer pair (the amplicon size was 100 to 150 bp) on a LightCycler (LightCycler 480, Roche), following the protocol of the manufacturer. Both actin and alg9 were employed as reference genes for quantitative PCR analysis. Standard curves were generated for each primer pair to estimate their amplification efficiency using the LightCycler software (LightCycler 480, Roche), and the quantitative PCR data were accordingly adjusted for use in subsequent analysis. The theoretical amplification efficiency is 2.0, and the actual amplification efficiencies of the different primer pairs were 1.97 (gfp), 1.89 (npabgs), 1.98(actin), 1.93 (alg9), 1.94 (egIII), 1.98 (cbhI), and 1.98 (kan).

### Quantitative assays of enzyme activity

The supernatants collected from yeast cultures were prepared for cellulase activity assays. The commercial cellulolytic enzyme mixture kits of 0.5 unit Celluclast 1.5 L (Novozyme, Denmark) and 1 unit Novozyme 188 (Novozyme, Denmark) were used as benchmarks. The commercial and yeast produced enzymes were assayed at the same MUC activity at 30°C with different protein concentrations, such as Celluclast 1.5 L (0.03 mg/ml), Novozyme 188 (0.85 mg/ml), and supernatant of yeast culture (1.3 mg/ml). The total glucanase activity was assayed by adding 40 μl of supernatant to final 100 μl of buffer solution (0.8 mg/ml 4-methylumbelliferyl-β-D-cellobiopyranoside (MUC), 0.05 M sodium acetate, pH 4.5) at 30°C for 1 hr. The enzyme activity of released 4-methylumbelliferone (MU) was measured in fluorescence units (FU) by the fluorescent intensity reader (SpectraMax M2, MDS) with excitation and emission wavelengths at 365 nm and 465 nm. The relative activity of phosphoric acid-swollen cellulose (PASC) digestion was assayed by mixing 40 μl of supernatant with 60 μl of buffer solution containing 0.4% PASC. Both of them were reacted in the 0.05 M sodium acetate, pH4.5 at 30°C, 24 hrs. After the hydrolysis reaction, the amount of reducing sugar was measured using the Somogyi–Nelson method to determine the number of glucose equivalents [32]. The relative activity of endo-glucanase was assayed by mixing 40 μl of supernatant with 60 μl of buffer solution containing 0.4% (w/v) Azo-CM-Cellulose (Dye-CMC) (Megazyme, Wicklow, Ireland) at 30°C, 6 hrs, and the detection was done via absorption of 590 nm. To quantify the β-glucosidase activity, 10 μL of the yeast culture supernatant was added to deep-well microtiter plates with each well containing 90 μL of 50 mM p-nitrophenyl-β-D-glucopyranoside (pNPG) (Sigma-Aldrich, St Louis, MO, USA), 0.05 M acetate buffer pH 5.0, at 30°C, 10 mins. The detection was done by the fluorescent intensity reader with absorbance at 410 nm. The protein concentration was determined by the Bradford method.

### Carbon source utilization and ethanol production assay

The transformed yeast cells were grown on 2% agar YP medium plates with cellobiose, β-glycan, CMC, or PASC as the single carbon source. This recipe in liquid medium was also used for yeast growth and ethanol fermentation. The semi-anarobic batch culturing was conducted in a 15 ml serum tube with 5 ml of medium with 10 ml of air in the bottle at 37°C, 120 rpm rolling. The initial inoculated cell density for each sample had an O.D. of 20 at a wavelength of 600 nm using a spectrophotometer (Ultrospec 2100 pro; Amersham Bioscience). Total sugar concentration was measured as glucose equivalent by the phenol-sulfuric acid method [29]. The productivity of ethanol was analyzed by gas chromatography (Shimazdu, GC-14, Japan) with a flame ionization detector (FID) and a stainless steel column (80/120 Carbopack B/6.6% Carbowax, 2 m x 2 mm), with nitrogen as mobile gas. The running condition included heating of the column from 80 to 150°C at a ramp rate of 4°C per min, an injection temperature of 180°C, and a detection temperature of 250°C. Each fermentation experiment and the subsequent analysis were repeated three times.

## Abbreviations

PGASO: Promoter-based Gene Assembly and Simultaneous Overexpression; CBP: Consolidated bioprocessing; MUC: 4-methylumbelliferyl-β-D-cellobiopyranoside; PASC: Phosphoric acid-swollen cellulose; CMC: Carboxymethyl cellulose; Dye-CMCAzo-CM-Cellulose; pNPG: p-nitrophenyl-β-D-glucopyranoside.

## Competing interests

The authors declare that they have no competing interests.

## Authors’ contributions

J-JC and C-Y H designed experiments. J-JC, C-YH, F-JH, T-YT, H-MK, H-LC, and CH-TW carried out the experiments, analyzed the data and drafted the manuscript. W-HL, C-CH and M-CS supervised the study. W-HL and C-CH revised the manuscript. All authors read and approved the final manuscript.
